# Woman-centred care during pregnancy and birth in Ireland: thematic analysis of women’s and clinicians’ experiences

**DOI:** 10.1186/s12884-017-1521-3

**Published:** 2017-09-25

**Authors:** Andrew Hunter, Declan Devane, Catherine Houghton, Annmarie Grealish, Agnes Tully, Valerie Smith

**Affiliations:** 1 School of Nursing and Midwifery, National University of Ireland, Galway, Galway, Ireland; 2 Florence Nightingale Faculty of Nursing & Midwifery, King’s College London, London, United Kingdom

**Keywords:** Women-centred care, Choice in childbirth, Qualitative enquiry, Framework analysis, Thematic analysis

## Abstract

**Background:**

Recent policy and service provision recommends a woman-centred approach to maternity care. Midwife-led models of care are seen as one important strategy for enhancing women’s choice; a core element of woman-centred care. In the Republic of Ireland, an obstetric consultant-led, midwife-managed service model currently predominates and there is limited exploration of the concept of women centred care from the perspectives of those directly involved; that is, women, midwives, general practitioners and obstetricians.

This study considers women’s and clinicians’ views, experiences and perspectives of woman-centred maternity care in Ireland.

**Methods:**

A descriptive qualitative design. Participants (*n* = 31) were purposively sampled from two geographically distinct maternity units. Interviews were face-to-face or over the telephone, one-to-one or focus groups. A thematic analysis of the interview data was performed.

**Results:**

Five major themes representing women’s and clinicians’ views, experiences and perspectives of women-centred care emerged from the data. These were Protecting Normality, Education and Decision Making, Continuity, Empowerment for Women-Centred Care and Building Capacity for Women-Centred Care. Within these major themes, sub-themes emerged that reflect key elements of women-centred care. These were respect, partnership in decision making, information sharing, educational impact, continuity of service, staff continuity and availability, genuine choice, promoting women’s autonomy, individualized care, staff competency and practice organization.

**Conclusion:**

Women centred-care, as perceived by participants in this study, is not routinely provided in Ireland and women subscribe to the dominant culture that views safety as paramount. Women-centred care can best be facilitated through continuity of carer and in particular through midwife led models of care; however, there is potential to provide women-centred care within existing labour wards in terms of consistency of care, education of women, common approaches to care across professions and women’s choice. To achieve this, however, future research is required to better understand the role of midwife-led care within existing labour ward settings. While a positive view of women-centred care was found; there is still a difference in approach and imbalance of power between the professions. More research is required to consider how these differences impact care provision and how they might be overcome.

## Background

Internationally, the last 15 years have seen policy makers and service providers recommend that maternity services become more woman-centred [1–3]. A key component of these recommendations is the promotion of additional models of maternity care including midwife-led models of care which are seen as one important strategy in enhancing women’s choice, a key component of woman-centred care (WCC). In the Republic of Ireland (ROI), however, choice in models of maternity care has not kept pace with that evident in services in other high income countries. Currently, an obstetric consultant-led, midwife-managed service model predominates [4]. This model involves the vast majority of women giving birth in urban maternity hospitals, including over one-third of all women giving birth in one of three urban maternity hospitals, under the lead care of a consultant obstetrician with midwives. It is hoped however that Ireland’s first national Maternity Strategy [5], published recently on foot of significant shortcomings in Irish maternity services, will offer more choice of quality models of maternity care to women and their families.

In the United Kingdom (UK), reports into the nature of maternity services in the 1990s [6–8] resulted in a change of emphasis towards WCC. This policy driven change directed service providers to engage with and meet the needs of women, placing them at the centre of planning and evaluation maternity services. In the ROI, the absence of a cogent national maternity strategy has, until recently, facilitated the continuation of a largely singular model of maternity care, that is, a consultant-led, midwife-managed service [9]. Consequently, there has been limited development of midwife-led models of maternity care other than small, albeit important, isolated clusters including the establishment of two midwife-led units [10]. The ROI continues to offer an obstetrician led approach to care similar to the approaches in Iran and Lebbanon [11]. This is in spite of a growing body of evidence from other high income countries such as the UK, France, Australia and New Zealand where evidence led alternative approaches such as midwife-led continuity of care have been adopted [11]. Implementation of midwife led approaches emphasizing WCC have been shown to offer equivalent levels of safety with the added benefit of giving women more choice resulting in more positive pregnancy, birth and post-natal experiences [10, 11].

The recent National Maternity Strategy (2016) calls for an increased choice in models of maternity care in Ireland [5]. One clear barrier to change is a lack of clarity on stakeholders (women and clinicians) understanding and knowledge of what constitutes WCC, and a lack of evidence that gives a qualitative voice to women’s views, experience and preferences while availing of current maternity services [10, 11]. For this reason, there is a need to describe the core attributes or indicators of WCC during pregnancy, birth and postnatally in Ireland, from the perspective of all stakeholders; that is, women, midwives, general practitioners and obstetricians, and, in doing so, achieve a better understanding of WCC which will provide an important foundation for developing strategies for change.

## Methods

### Aim

The aim of this study was to explore the concept of WCC during pregnancy and birth within the Irish context, and, through women’s and clinicians’ views, experiences and perspectives, identify the key elements of WCC so that it might be better understood.

### Study design and setting

A descriptive qualitative study design was chosen as the most appropriate design to address the research aim. This design presents and interprets participant data in a rigorous and clear manner [12] utilizing methods best suited to the area of interest. In this instance, data were collected via face to face or telephone interview and focus group. Framework and thematic analysis were then utilized to analyze the data. To explore potentially common and unique manifestations of WCC, two geographically distinct maternity units were chosen from which to access the stakeholder groups. These were a large urban maternity hospital (circa 8000 births per year) and a regional maternity hospital (circa 4000 births per year), both with similar philosophical and organizational approaches, that is predominantly consultant-led, midwife-managed care, with, to a lesser extent, embedded or alongside midwife-led care.

### Ethics

Ethical approval for the study was granted by the research ethics committees of the two participating hospitals. Written consent to contact was obtained by the gatekeepers and participant contact details were forwarded to the interviewer who subsequently contacted participants to arrange a suitable date, time and venue for the interview. The purpose of the research was further explained to all prospective participants, including potential benefits and harms, and, they were asked if they were willing to take part. All participants being interviewed provided written consent prior to commencing the interview.

### Sampling and data collection

The participants (*n* = 31) were purposefully sampled from diverse stakeholder groups (Table 1), providing a range of perspectives and knowledge of WCC. Two midwives, working at the study sites, acted as gatekeepers, and assisted with the recruitment process by advertising the study locally, by distributing the study information, verbally and in written format, and by discussing the study further with eligible participants. Although the study did not seek to compare models of maternity care or views/experiences of different models of maternity care directly; participant perspectives on what does or does not comprise WCC across the available settings (consultant-led care only, midwifery-led care only, and consultant and midwifery-led care) was purposively sought so as to ensure stakeholders working in the various settings were represented in our study sample. The majority of participants were interviewed individually, in person or by telephone, by the same researcher (VS). Focus group interviews were conducted with single professional groups; that is, two focus groups with midwives (*n* = 4) only and one with GP participants only (*n* = 5). This approach was necessary for reasons of access, logistics and practicality. Collecting interview and focus group data arising from discussion amongst participant groups added to the variation and depth of the overall data. The mean interview duration was 32 min (range 21–45 min). All efforts were made to ensure participation from all members of the groups and the focus group participants were asked the same questions as the individual interview participants, thus ensuring a pragmatic and consistent approach to data collection [12]. To ensure baseline consistency across interviews and groups, an interview schedule, based on four broad questions, was used to guide the information sought. These broad questions were (i) what do you understand by women-centred maternity care?; (ii) what do you think are the important components of women-centred maternity care?; (iii) what things can/do maternity services do that engenders women-centred maternity care?; and, (iv) what things should maternity services avoid or minimize in promoting women-centred care?

**Table 1:** Study Participants

Participant Type	Number (Site 1/Site 2)^a^
Women (postnatal)	11 (4/7)
Midwives	10 (5/5)
Obstetricians	5 (2/3)
General Practitioners	5 (1 GP practice)

^**a**^ Site 1 = urban maternity hospital; Site 2 = regional maternity hospital

The interviews were conducted from January to April 2015

## Data analysis

Qualitative data analysis, following verbatim transcription of the interviews and focus groups, was conducted by a team of four researchers (AH, CH, AG, AT) who were not involved in conducting the interviews. All of these researchers are nursing academics and have personal experience of the Irish maternity care system. All interviews were audio recorded, anonymized to ensure confidentiality and transcribed verbatim by professional transcribers and checked by the research team for accuracy. Thematic analysis was used incorporating a framework analysis approach [13]. Framework analysis [14] has five key stages (familiarization; identifying a thematic framework; indexing; charting & mapping and interpretation). The first two stages were used in this analysis to help guide the analysis team, that is, familiarization and identifying a thematic framework.

Familiarization involved two members of the team developing coding themes from two of the transcripts in order to become familiar with the data. In stage two, all four members of the team then coded a single transcript using the framework. This process allowed the team to acknowledge their own personal perspectives, modify the framework and develop inter-coder agreement. Coding stripes within NVivo were used to determine the level of agreement between team members [15]. This process allowed comparison, discussion and agreement of the framework for stage three.

In the third stage of analysis, each team member was assigned a number of transcripts to code. Annotations were used to document researcher questions, decision about the data, reflections on analysis and to provide a clear audit trail of the process. The themes and their titles were subject to restructuring from the original framework as similarities and differences were identified allowing accurate representation of what participants were saying (see Table 2). At this stage, NVivo was utilized to apply query tools to check the accuracy of the themes and associated memos. The final stage of analysis involved using executive summary statements as the foundation for drafting the results that described the views, perceptions and experiences of women, midwives, obstetricians and GPs on the concept of WCC.

**Table 2:** Completed Thematic Framework

Protecting normality	• Normalising • Respect • Public perceptions
Education and decision making	• Decision making • Information sharing • Educational impact
Continuity	• Continuity of service • Fragmented care • Staff continuity and availability
Empowerment for WCC	• Genuine choice in WCC • Lack of choice • Promoting women’s autonomy • Individualised care
Building capacity for WCC	• Not providing WCC • Staff competency • Practice organisation

## Rigour

In this research, a number of strategies were used to ensure rigour. Firstly, triangulation was achieved through team analysis utilizing four researchers. In addition, the use of coding stripes within NVivo ensured inter-researcher agreement [15]. Multiple participant types ensured many perspectives were captured enhancing the completeness of the data, which is another important element of triangulation [16]. The ability to write annotations in NVivo facilitated transparent decision making processes between the four researchers, reduced bias and provided an audit trail supporting the results [17]. Query tools such as matrix and coding queries were also used. NVivo was able to locate all the passages in the data ensuring that any themes and subsequent results could be checked and were supported by data from more than one individual participant [17]. Furthermore, matrix-coding queries provided a comparison of multiple nodes as a numeric table for illustrative purposes [18].

## Results

Five main themes emerged from the analysis of data within which there were sub-themes that highlighted key elements of WCC. The five main themes were: Protecting Normality; Education and Decision Making; Continuity; Empowerment for WCC; and Building Capacity for WCC. These results describe the views, perceptions and experiences of women, midwives, obstetricians and GPs on the concept of WCC.

### Protecting normality

This theme describes the finding from the data that pregnancy should be viewed as a normal function, not illness. In doing so, it reflects how respect for women and the philosophy of WCC can help to normalize the pregnancy journey regardless of the outcome. The sub-themes identified were: normalizing and respect.

All participant groups identified the importance of normalizing pregnancy and childbirth; with the nature of the pregnancy impacting on the health care professionals’ ability to do this. For instance, it was identified that normalizing pregnancy is more likely in midwife-led units (MLUs) because, by their nature, the women attending have more ‘straightforward’ pregnancies. A number of strategies were identified that help to normalize the pregnancy journey. These included respecting women’s wishes and following their birth plans. This midwife datum represents the view that the theme *protecting normality* relates to giving women choice and avoiding intervention where possible:

“*The way you can do that [normalizing] is by not doing things to women that they don’t need done to them, you know”* (Midwife-3)

These participants made an explicit link between Protecting Normality, specifically normalizing pregnancy and having care provided in smaller more accessible settings. This obstetrician participant echoes this view of normality, identifying the need to perceive pregnancy as a normal journey:

*“And this is important for the women to understand… that the pregnancy is not a disease, it’s not a special condition, it’s part of the normal healthy life”* (Obstetrician-3)

This view is supported by other participants who stress the importance of the environment in normalizing childbirth. This midwife notes the challenge faced in providing peaceful and quiet environments in busy, and potentially distracting, labour wards:

*“You’d be trying to create the right environment so lights really low, nice music on, the lavender going … it’s quiet, it’s dark, it’s peaceful... Where it’s [in the labour ward] bright lights and it’s busy and there’s noise”* (Midwife-5)

Decentralizing the provision of services was identified as a means of protecting normality. This participant indicates that it is difficult to normalize pregnancy when services are inaccessible to women:

*“If we valued women truly then we wouldn’t make them drive sixty miles to the nearest unit”* (Midwife-1)

A theme that emerged from the data and supported the concept of *protecting normality* was respect: respect for women, for the journey of pregnancy and for WCC. The women participants identified that respect meant being recognized as an individual with past experiences, preferences and potential fears about childbirth. These women participants illustrate the importance of feeling listened to as a component of respect:

*“you’re looking after the woman as well as just the mother of the baby …the woman is not just the mother like you know, she’s still just you know, like a person aside from being a mother”* (Woman-2)

*“Sometimes it’s nice to feel that you’re listened to and that you’re unique”* (Woman-5)

The midwife participants recognized the need for an ethos of respect. Importantly, this did not just refer to the outcome of pregnancy, but a respect for all stages of pregnancy and fertility. This datum identifies the need to establish a philosophy that normalizes and respects WCC, with midwives themselves having a role to play in implementing this philosophy into the care they provide:

*“Midwives need to change their way of thinking to provide the opportunity to women to have normal births and normal birth environment. So, I sometimes find it just a little bit frustrating that people will put up all sorts of objections… the doctors won’t let us? When I think it’s the midwives not letting themselves… and not standing together. And that’s what would help achieve.. you know normal, natural labour and birth”* (Midwife-2)

Respect for the principle of WCC was influenced by a range of societal factors, media influences and cultural beliefs about pregnancy. Professional participants consistently noted that a normal, safe pregnancy and birth could be accommodated within a MLU, with the proviso that there is a clear route to obstetric care and a labour ward should complications arise. What became clear from the data was that women themselves were unaware of this option due to the prevalence of the labour ward model in Ireland:

*“it’s [awareness of MLUs] not still getting out into the community, into you know that there is another alternative to going to have your baby in the hospital. ”* (Midwife-4)

This lack of awareness of alternatives is also indicated when participants view greater levels of medical intervention, not only as the norm, but also as a safer option resulting in better outcomes. These following quotes; first from a midwife and then an obstetrician, indicate professional openness towards change along with the challenge faced:

*“They (women) will always want to have the most high tech, and I think that will last for a long time, until the service is much more balanced, and the service and choices offered to women are more balanced. It will take a long time for the fear factor to disappear and for the education that comes from providing a fully rounded service actually pervades women’s psyche and they realise actually I don’t need every single scan.”* (Midwife-1)

*“Well I think I would imagine that you know if it is explained to women what this (WCC) is, you know what the advantages are, that they would see that.”* (Obstetrician-1)

While supportive of the principle of supporting WCC via the introduction of MLUs across Ireland this obstetrician goes on to articulate the status quo regarding risk and choice:

*“There’s an assumption that something had happened, or there’s bad publicity, or that you feel safer in going to urban. You know so it was seen as a continuum, and that you know if you are low risk care you go to your local hospital, the higher risk, a certain amount can be handled in your local hospital with input maybe from the tertiary centre, and then you have people who need the tertiary level care, full stop.”* (Obstetrician-1)

In summary, *protecting normality* identifies that participants believe pregnancy and childbirth can and should be perceived as normal, also that there should be a minimum of intervention in support of this normality. While all of the participant groups aspired towards this they were also clear that the current systems with emphasis on safety and the timely processing of women in labour wards meant that there would need to be significant change if normality is to be re-instituted in the face of near ubiquitous medical intervention. Inherent in any approach to protecting normality is the need to educate women and respect women’s primacy in decision making during pregnancy via a WCC philosophy.

### Education and decision making

The *education and decision making* theme describes how professional training impacts upon professionals’ education of women, affecting the decisions made across the journey of pregnancy. This theme brings together three sub-themes that emerged from the data: partnership in decision making; information sharing and educational impact. There was considerable agreement amongst the participants regarding the need to improve the education of women regarding their care options. The professional participants indicated that providing better quality education to women could improve the overall quality of WCC. Professional participants also provided examples of where their own education required improvement to allow more skilled educational support for women and to improve inter-professional understanding.

The sub-theme partnership in decision making reflects the understanding that WCC requires the provision of genuine choice through education, which can only be provided when stakeholders have knowledge regarding care options and when such care options are actually available. A view illustrated by this GP participant who noted the need to educate women on their options:

*“if you empower them (women) with all the information…, risks and benefits of certain treatments and ultimately you know, if they’re well informed, women can make their own decision about what kind of management they want. So I guess it’s trying to give them all the information so they can make decisions themselves about their own management. ”* (General Practitioner-5)

The information sharing sub-theme describes the need for and potential benefits of professions improving understanding of each other and being educated in each other’s approaches to care. This midwife participant makes the case for different stakeholders having a common skill set:

*“we need to introduce something called an obwife (laugh) instead of an obstetrician in isolation, not a midwife, maybe an obwife... I think the problem is nobody is the enemy here, the obstetrician is not the enemy, the women, the midwife is not the enemy but the poor woman should not fall between 2 egos. The women should get the care based on the best.”* (Midwife-10)

This datum reflects the view voiced across participant types that there was a need for greater inter-professional understanding and emphasis on a shared ethos of WCC. The educational impact sub-theme provides additional understanding by relating the information women are given to professional knowledge, organizational systems (in the example below whether the woman was consistently seen and comprehensively assessed) and individual confidence to provide individualized rather than generic advice. This midwife participant illustrates the range of components that must be in place along with education to ensure that decision making is woman-centred:

*“co-sleeping is one example, I would find it very hard to say to a mother not to co-sleep with her baby in the first 8 weeks. But as a professional I have to be aware of giving her the correct information. And I think currently the correct information is not to co-sleep. But I would find that very hard. But I cannot do a disservice to the woman because I don’t know if her bed is big enough. I don’t know if her home is properly heated, if her partner or herself smokes or takes recreational drugs or takes any prescribed medication.”* (Midwife-1)

The theme *education and decision making* represents the finding that professionals need to develop their own knowledge, along with developing a shared ethos of pregnancy and child birth. The lack of a shared ethos is currently viewed as limiting choice by women and for women and as a barrier to WCC. Raising inter-professional knowledge was viewed as a means of enhancing access to other models of care as trust between the professions was seen as a requirement for change.

### Continuity

The theme continuity brings together three sub-themes: continuity of service, fragmented care, and staff continuity and availability. Firstly, continuity of service, relates to the importance of having not only continuity of care during pregnancy but also in labour and in the post-natal period. Consistency of clinician over the term of pregnancy, that is, continuity of carer, was considered synonymous with good quality care. One obstetrician interviewed noted that women express dissatisfaction with hospital services where women tend to see different doctors, and do not view themselves as being offered care by a consistent team:

*“...if we could manage to be a little more consistent regarding who is getting seen, or that there is a smaller group of people that might see the woman, so that she is getting the impression that she’s been seen by a team rather than by nobody in particular. ..I think that’s about consistency”.* (Obstetrician-1)

All participants noted that MLUs provide greater continuity of carer. This midwife participant, based in a labour ward, articulates their view of continuity as a potential benefit of a MLU service:

*“if you look at other models of care then like midwife-led clinics, maternity, midwife-led units, where you have smaller teams, you have a chance of obtaining continuity of carer”* (Midwife-7).

The sub-theme, fragmented care, extends the understanding of the implication of inconsistent service provision. This GP participant notes how different professionals are involved in caring for the pregnant woman and how they tend to work parallel to rather than communicating effectively with each other. In this example, they view failures in communication as fragmentation:

*“[I have] had patients who, em, have attended a hospital with a miscarriage but haven’t come through you so you’re not aware and then they arrive in a couple of weeks later and you say oh you’re in for your check-up. And they say well actually no”* (General Practitioner-5).

This midwife views service fragmentation as a feature of having three layers to the perinatal service; antenatal, labour and postnatal care:

*“the current service does an awful lot to prevent continuity of care, in terms of… the organization of our services, we have antenatal, we have labour ward, we have postnatal, we have fragmented what is a continuous process of pregnancy…if you look at obstetricians, midwives, GPs, public health nurses…we all function again almost independently or separately from each other... So the first thing you have to do is prioritize it, because all the literature would say it was important”* (Midwife-6).

The staff continuity and availability sub-theme emphasized the importance of relationship building between women and professionals. These midwife participants viewed this as maintaining the woman at the centre of care:

*“[The MLU is] excellent in involving women a lot more and I think the fact that they tend to see the, say midwife, or generally speaking see the same midwife for each hospital visit has been huge... they seem to discuss a lot more, and they seem to develop a very, or foster a very good relationship between the midwife and the, the woman”* (General Practitioner-5)

The continuity theme highlights the need for women to have ease of access to services, good communication between service providers and consistent contact between women and staff. In practice, much care is noted to be fragmented with care being received in parallel and limited communication between primary care, hospital services and MLUs.

### Empowerment for WCC

This theme illustrates how empowerment is viewed as a precursor of WCC and was valued highly and regarded as important for women across the pregnancy journey. This theme also describes how empowerment can have an impact on women’s choice and autonomy. This theme comprises four main sub themes; that is, genuine choice in WCC, lack of choice, promoting women’s autonomy, and individualized care. There was considerable consensus amongst the participant groups regarding the importance of choice and how this could be facilitated but the data also indicated the lack of choice and resultant lack of WCC.

The sub theme, genuine choice in WCC, describes how resources in maternity care are linked with lack of choice in the birth plans and flexibility in terms of location of care. This woman participant highlights the conflict between existing service provision and a woman focused service offering more choice:

*“I suppose its focusing on the woman’s care and the baby as opposed to what a clinical led care, you know you’d have a say in what you would like, you know what women would want themselves for their maternity, what they feel, I presume that’s what it means”.* (Woman-3)

The sub theme, lack of choice, describes how limited the services are when it comes to flexibility and how participants identify that enforced changes to daily routine are also related to lack of choice:

*“And even the diabetic ladies coming in and they say well I don’t usually take my insulin till ten when I’m at home. But they’re getting their breakfast at eight o’clock here and then they’re saying their blood sugars are out because she’s not in her normal routine”.* (Midwife-10)

The sub-theme ‘promoting women’s autonomy and empowerment’ describes the participants’ experiences of power, control, knowledge and their influence upon informed decision making. The participants provided examples of the importance of listening, partnership working and shared decision making which places women at the centre of their care:

*“...you go for your antenatal visits and you’re kind of like powering through the system in five minutes and out the door again, you’re kind of, you walk by feeling there’s no one really listening to you, you don’t feel they care……. you know it would be nice to have that sort of, feeling that someone actually is listening to you and kind of cares what happens to you, it’s the woman as well not just the baby”*. (Woman-5)

The final sub theme ‘individualized care’ describes how important communication is in sharing of information and partnership working, and providing emotional and practical support in order that informed choices can be made. This was strongly stated by the women participants, with the following two quotes illustrating different aspects of ‘individualized care’:

*“I suppose it’s because you’ve never looked after, a hundred percent of the time, a newborn infant before, so it just gives you a heads-up, and they show you how to care, how to bathe a newborn, how often to feed them. Nothing can prepare you for holding that newborn in your arms, but it does go some way towards helping. And as well you get to meet other women who are in the same boat as you, and you get to, you know, chat with them as well”*. (Woman-1)

*“And they were saying relax, everything is fine, you’re doing perfect, you’re doing great. And they just talked me through so, it was just the easiest labour I ever had with this specific midwife I had at that time. And that was definitely woman centred care. You know she was there with me, she was talking through with me, you know she was telling me everything was fine, we’re here, we have everything, you know don’t worry, nothing else is going to fall out (laugh)”.* (Woman-5)

The theme Empowerment for WCC describes how providing a woman with choices is a key principle of maternity care and the lack of empowerment diminishes WCC. All of the participant groups were generally critical of the failure of services to empower women in current Irish maternity services. Participants identified limited resources, lack of choice over location of care, and clinician’s’ application of rule structures within the services as inflexible, hierarchical and disempowering to both women and professionals. This theme also represents a consensus between the participant groups on the importance of individualized care, the need for better communication, sharing of information and partnership working. Empowerment may also be enhanced by providing emotional and practical support to facilitate informed choice.

### Building capacity for WCC

The theme *building capacity for WCC* describes how the data indicated that WCC can be developed and maintained. This theme comprises three main sub-themes: not providing WCC, staff competency and practice organization. Participant data indicated some instances where WCC was achieved, with the majority of the data indicating a lack of capacity for WCC.

The sub-theme, not providing WCC, describes how limited resources in maternity care, linked with existing ways of working, impact negatively on overall capacity to provide WCC. This links *building capacity for women WCC* with other themes specifically continuity as illustrated by this GP datum:

*“They’re kind of having to retell their story I think each time that they go in for this (outpatient appointments). There’s a lack of continuity there I think”*. (General Practitioner-2)

These data are consistent with other data indicating a preference for, but lack of continuity of carer. The data also demonstrates that the organizational structure and emphasis on safety within stretched labour ward environments limits WCC:

*“one midwife to fourteen mums and fourteen babies, if you eventually get to the stage where you can mention anything about those things [general care such as varicose veins or sciatica], then you are doing amazing in that environment. So, yea, I think the art is getting squashed out for us, and we could do more for women”* (Midwife-1)

The second sub-theme, staff competency, refers to the ability of professionals to work with women in a manner that provides a range of choices. Communication, both with women and inter-professionally is shown to impact upon the provision of WCC. This midwife datum suggests that where there is a lack of common ethos and understanding between the professions, particularly obstetricians and midwives, it can impact on quality of care reducing WCC:

*“nobody is the enemy here, the obstetrician is not the enemy, the women, the midwife is not the enemy but the poor woman should not fall between 2 egos. The women should get the care based on the best evidence.”* (Midwife-4)

While organization and resources are often noted as impacting on capacity, staff experience is also indicated. This midwife participant notes that individual experience, professional knowledge and confidence can add to the potential for WCC to occur:

*“But then you know the levels of experience and what levels then do you allow to make those decisions, would be up for, that’s another discussion in itself, who makes the final management plan? Because like you might have someone who has years of experience and they have the knowledge that they can push a woman that bit further in order to maintain normality and a safe outcome. As opposed to someone else who might be having fear of well we can’t push her, let’s deliver”* (Midwife-6)

In addition, analysis of these data indicate that WCC arises from the capacity to provide women with a range of informed choices. Importantly, these choices need to be underpinned by common ethos and capacity across professions, as summed up by this datum:

*“And like that I don’t feel that it’s always going to be midwifery based, someone with a history of two previous sections, knows she’s not going to have a normal delivery. But what’s the safest way for her; I think it’s about safety and informed choice”* (Midwife-6)

An opposing view is presented by this obstetrician participant who suggests that women’s choice is and should be limited on practical grounds:

*“So if you are too liberal with the woman deciding the choice, it’s hard to work it (an outpatient clinic) in the way that our system works currently”* (Obstetrician-1).

That the obstetrician refers to ‘current’ systems reflects the understanding, prevalent within the data, that achieving WCC will require significant change and investment. The final sub theme, practice organization, describes how current organizational structures can serve to diminish or enhance WCC. This midwife compares public and private care suggesting that private care can offer more continuity and therefore be more woman-centred:

I believe that women, a lot of women, they buy private maternity care, they probably do it to get a certain amount of nicer accommodation, even during the pregnancy, less waiting times – but they buy continuity of carer. And that must be extraordinary for them to be able to go to the same person again and again and again. And the sad thing about our services is you have to pay for it out of your own pocket, every woman should have the chance of that within the public healthcare system. (Midwife-2).

The idea that current labor ward organizational environments reduce capacity for WCC is also referred to in terms of staff-woman interaction. The ratio of midwives to women and the ethos of ensuring rapid turnover is referred to regularly in the data:

*“When you’re in you’re in and out in 5 and 10 minutes. However if you attend a midwifery led clinic you know there might be, let’s say 15 women attending one midwife as opposed to 140 women attending 3 obstetricians. And like midwives will give women time and give them the opportunity to ask questions and all of that”* (Midwife-5)

In summary, *building capacity for WCC* describes how WCC is currently negatively impacted by a range of factors including organizational structures, professional differences in ethos, experience and skills. While this data is largely negative, there is a common view that there is a need to raise capacity, specifically the degree to which women are offered choice in models of maternity care and continuity of carer.

## Discussion

This study set out to explore the views and experiences of women and clinicians of WCC during pregnancy and childbirth in the ROI so that key elements of the concept might be highlighted and better understood. The analysis indicates themes that could potentially inform the achievement of WCC. The interaction of these themes: protecting normality, education and decision making, continuity, empowerment for WCC and building capacity for WCC indicate strategies towards WCC. These identified themes resonate, in part, with findings from previous studies on WCC. For example Maputle and Donavon [19], in their concept analysis of WCC, identified information sharing and empowerment as key attributes of WCC. They also identified participative decision-making, informed choices and autonomy, and open communication and respect as antecedents to WCC. Others [20, 21] assert how WCC incorporates continuity of care, control over care decisions, and choice in all aspects of care.

The findings of this study illustrate how WCC could potentially be achieved, but are clear that the participating professionals did not view the care they provide as woman-centred as it ought to be/should be. In addition, the data from women participants indicates that, while they share preferences for some of the same constituents of WCC as professionals, these women have little experience of a maternity service that is truly women-centred. This finding is similar to that identified in other Irish and international studies [10, 11, 22], that note women often do not request, recognize or value WCC due to a lack of knowledge. In spite of this finding all of the professional participants indicated that the majority of women experiencing uncomplicated pregnancies should be offered a midwife led service, viewing this as a means of enhancing WCC and providing women with choices.

The results show a continued lack of alternatives to the prevailing obstetric consultant-led model of care in the ROI. The professional participants indicated that they aspired to WCC, but due to organizational and educational challenges do not routinely provide it. In addition, the prevailing consultant-led model of care provided in labour wards in the ROI continues to limit the ability of professionals to offer the full gamut of WCC. The midwife participants indicated the existence of a skill, power and decision making hierarchy. This hierarchy is reinforced through an emphasis on safety, and acts as a major challenge to the provision of WCC. The hierarchy identified in this study is not unique with similar results reported in other research [23, 24]. In this study, participants placed the decision making influence of doctors ahead of midwives, with women themselves subordinate to all professional decision making. Women participants, valued some aspects of WCC such as choice and normalizing childbirth, but also equated medicalized care with normality and associated this with safety. In practice, women participants lacked alternative normalities to that of medical labour wards as they had not given birth within any other organizational structures. Therefore, women routinely equated successful contact with services to having a healthy baby born under medical supervision, rather than having other alternatives in mind or considering the overall quality of their experience. Discussion around the policy direction of pregnancy and childbirth in Ireland [3] talks of the ‘long road’ away from medically led and intervention focused maternity care in Ireland. This study, along with others [4, 24] considers the experience of maternity care and childbirth in the ROI, and indicates that the long journey continues.

### Strengths and limitations

This study is strengthened by the inclusion of multiple stakeholder groups (women, midwives, obstetricians and GPs) and aggregating data from interviews and focus groups, thus offering varying perspectives and knowledge of WCC for informing the collective results. The inclusion of stakeholders from two hospital locations may have limited the overall findings, however, we believe this was minimised by choosing diverse study sites, that is one urban and one regional, where care, in the main, is reflective of mainstream Irish maternity care. Similarly analysis of data derived from different methods may have limited the findings, however we believe it provides a depth of aggregated collective findings based on variation in data collection methods.

### Implications for practice and research

There is now a body of research evidence and policy guidance calling for change in the manner in which pregnancy care and childbirth services are delivered [4, 25, 26]. In spite of these calls, the complex hierarchical systems experienced by all participants in this study mean that WCC is not routinely provided and technical and medical approaches to care continue to dominate in many settings [23, 24, 26].

Research [4, 11] indicates that midwife-led models of care can offer a service, which is, at least, as safe as other models of care and results in in less intervention and increased WCC. The results from this study support those results that for many women WCC is best delivered via a midwife led model of care [4, 27]. In addition to supporting the need to develop more MLUs in Ireland our results indicate that there is potential to provide more WCC within existing labour wards while moving towards MLUs. WCC should not be the preserve of MLUs alone. In terms of continuity of care, education of women, common approaches to care across professions and women’s choice there is a need to shift labour ward practice towards more WCC. To achieve this, future research must better describe and understand the role of midwife led care within existing labour ward settings. Developing an understanding of the current standard model of care would provide a basis for change. Such an understanding could also direct changes in midwifery care as part of moves towards continuity orientated midwife led care within the wider multi-disciplinary team in support of WCC. In spite of the finding that participants in this study take a positive view of WCC and the potential for MLUs [4, 28]. There is still clearly a difference in approach and imbalance of power between the professions. More research is required to consider how these differences impact care provision and how these can be overcome.

Understanding women’s experiences and preferences also requires further research. As the recipients of care, women lack awareness of alternatives to medicalized care and their consistent view that medicalized care equates to safety and ‘normality’ was a key finding. Greater understanding of the impact of current care delivery systems, including the public-private imbalance, on the national understanding of pregnancy and childbirth is required to inform any changes towards WCC. Further studies are required to provide understanding of the degree to which women would like to be included in the decision making process and the impact such inclusion would have upon their pregnancy, birth and post birth experience. This will require the development of research approaches that assess women’s experience. Such methodological developments will have to consider the current low level of consultation and choice arising from the lack of WCC. Therefore, future methodological developments aimed at determining the psychosocial experience of women and impact of choice will have to include education of women in what these choices are and their implications.

## Conclusion

The results of this study indicate that irrespective of the renewed policy, practice and research emphasis on WCC provision as an important goal for service providers, the current reality is that WCC, as perceived by included participants, is not experienced by most women. In fact, the consultant-led model of care is so much the norm in the ROI that women are largely unaware of the possibility of alternative approaches. Professionals, while aware of alternatives, are so burdened by the maintenance of a safe service within an under resourced system that improving the experience of women via the provision of WCC will be challenging. The recent Irish National Maternity Strategy serves as the template for the ‘maternity workforce to … work together, in partnership across professions and with families, to deliver a new, better and safer maternity service’ ([5] p.3).

## Abbreviations

MLU: Midwifery led unit
ROI: Republic of Ireland
WCC: Women-centered care
